# Progress toward eliminating TB and HIV deaths in Brazil, 2001–2015: a spatial assessment

**DOI:** 10.1186/s12916-018-1131-6

**Published:** 2018-09-06

**Authors:** Jennifer M. Ross, Nathaniel J. Henry, Laura A. Dwyer-Lindgren, Andrea de Paula Lobo, Fatima Marinho de Souza, Molly H. Biehl, Sarah E. Ray, Robert C. Reiner, Rebecca W. Stubbs, Kirsten E. Wiens, Lucas Earl, Michael J. Kutz, Natalia V. Bhattacharjee, Hmwe H. Kyu, Mohsen Naghavi, Simon I. Hay

**Affiliations:** 1 Division of Allergy and Infectious Diseases, Department of Medicine, University of Washington, Seattle, Washington, USA; 2 Institute for Health Metrics and Evaluation, University of Washington, 2301 5th Ave Suite 600, Seattle, WA 98121 USA; 3 Department of Public Health, University of Brasilia, Distrito Federal, Brazil; 4 Department of Health Surveillance, Ministry of Health, Brasilia, Brazil

**Keywords:** Tuberculosis, HIV, Small area estimation, Geospatial, Geographic, Brazil, Case fatality, Mortality

## Abstract

**Background:**

Brazil has high burdens of tuberculosis (TB) and HIV, as previously estimated for the 26 states and the Federal District, as well as high levels of inequality in social and health indicators. We improved the geographic detail of burden estimation by modelling deaths due to TB and HIV and TB case fatality ratios for the more than 5400 municipalities in Brazil.

**Methods:**

This ecological study used vital registration data from the national mortality information system and TB case notifications from the national communicable disease notification system from 2001 to 2015. Mortality due to TB and HIV was modelled separately by cause and sex using a Bayesian spatially explicit mixed effects regression model. TB incidence was modelled using the same approach. Results were calibrated to the Global Burden of Disease Study 2016. Case fatality ratios were calculated for TB.

**Results:**

There was substantial inequality in TB and HIV mortality rates within the nation and within states. National-level TB mortality in people without HIV infection declined by nearly 50% during 2001 to 2015, but HIV mortality declined by just over 20% for males and 10% for females. TB and HIV mortality rates for municipalities in the 90th percentile nationally were more than three times rates in the 10th percentile, with nearly 70% of the worst-performing municipalities for male TB mortality and more than 75% for female mortality in 2001 also in the worst decile in 2015. The same municipality ranking metric for HIV was observed to be between 55% and 61%. Within states, the TB mortality rate ratios by sex for municipalities in the worst decile versus the best decile varied from 1.4 to 2.9, and HIV varied from 1.4 to 4.2. The World Health Organization target case fatality rate for TB of less than 10% was achieved in 9.6% of municipalities for males versus 38.4% for females in 2001 and improved to 38.4% and 56.6% of municipalities for males versus females, respectively, by 2014.

**Conclusions:**

Mortality rates in municipalities within the same state exhibited nearly as much relative variation as within the nation as a whole. Monitoring the mortality burden at this level of geographic detail is critical for guiding precision public health responses.

**Electronic supplementary material:**

The online version of this article (10.1186/s12916-018-1131-6) contains supplementary material, which is available to authorized users.

## Background

Brazil is a high-burden country for tuberculosis (TB) and human immunodeficiency virus (HIV)-TB co-infection [1] and also characterised by high levels of inequality in social and health indicators [2–4]. The twin slogans of ‘Leave no one behind’ and ‘Everybody counts’ adopted for World Tuberculosis Day and World AIDS Day, respectively, in 2017, emphasise the importance of reducing inequality to end these leading epidemics [5]. TB and HIV inequalities may manifest in geographic patterns because the underlying risk factors for TB and HIV infection and death, such as poverty, incarceration, undernutrition, crowding and poor access to health services, vary across geographic areas and through time [6–10]. Additionally, the disease mechanisms of transmission between persons in close contact can lead to geographic clusters of disease burden [11–13]. Brazil has invested in massive social programmes to improve health and equality, such as the Family Health programme of free community-based healthcare, the Bolsa Familia programme of cash transfers conditional on education and health behaviours [14], and universal eligibility for free TB care and free antiretroviral therapy for HIV infection since its discovery in 1996 [15]. The national strategy to end TB in Brazil prescribes TB control strategies based on local epidemiology; fine-scale mapping of TB and HIV burden can provide information to prioritise additional programmatic investments toward improving health [16].

Prior investigations of the spatial distribution of TB and HIV burden in Brazil varied in their scope and level of geographic detail, but few achieved coverage of the entire nation at fine spatial resolution or for long time series. The Global Burden of Disease (GBD) study collaborators modelled mortality due to an exhaustive set of causes, including HIV and TB, at the state level for 1990 through to 2015 [4]. Other investigations modelled mortality or case notifications at finer spatial scale for portions of the country [17–20]. Harling et al. [21] completed a nationwide municipal-level analysis of case notifications in Brazil of a shorter time series, 2002 to 2009. Outside of Brazil, there are few national-level spatial modelling studies of TB incidence and, to our knowledge, no nationally comprehensive spatial models of TB mortality at fine spatial scale [22–24]. There are broader spatial modelling efforts for HIV, corresponding to the greater availability of spatially resolved data sources for HIV than for TB in high-burden countries [25, 26].

There are methodologic challenges associated with spatial modelling of TB and HIV mortality which are addressed by this analysis. First, despite being leading infectious causes of death globally, TB and HIV death counts are low in small areas, leading to instability in case counts and difficulty in separating true differences in risk from stochastic noise for individual geographic areas. A modelling approach that draws strength from neighbouring groups across space and time could stabilise these estimates. Second, TB and HIV deaths may be misclassified due to failure to recognise the cause of death as HIV or TB or stigma associated with reporting these conditions [27–29]. Furthermore, the International Statistical Classification of Diseases (ICD) convention is for TB deaths in persons living with HIV infection (PLHIV) to be assigned to HIV as the underlying cause, which can hide the contribution of TB to these deaths if only a single cause of death is reported in vital registration [30]. In this study, we address these challenges by utilising comprehensive cause of death assignment and small area estimation to conduct a nationwide analysis of TB and HIV mortality at fine geographic scale. We also estimate the TB case fatality ratio, defined as the proportion of persons with TB who die of TB, a key metric in the World Health Organization (WHO) End TB Strategy [1]. HIV case fatality ratios are not estimated due to a lack of data to inform HIV incidence.

## Methods

### Overview

GBD collaborators estimated mortality burden for 249 causes of death from 1990 to 2015 for the 26 states and Federal District in Brazil [4]. This study extended the modelling of deaths assigned to TB or HIV by GBD 2016 to the second administrative level (municipality) using the municipality of residence recorded in vital registration records. TB incidence was modelled at the second administrative level using TB case notification records. All rates presented are age-standardised unless otherwise stated. This study complies with the Guidelines for Accurate and Transparent Health Estimates Reporting (GATHER; http://gather-statement.org). Analyses were done with R version 3.2.4 [31].

### Study design and data sources

This ecological study included all municipalities in Brazil. Mortality data included anonymised individual-level records from all deaths reported in the Brazil Mortality Information System occurring between January 1, 2001, and December 31, 2015. These records were tabulated according to the decedent’s municipality of residence, age, sex, and cause of death coded according to the tenth revision of the ICD (ICD-10), which was adopted in Brazil in 1996 (Additional file 1: Table S1) [30]. Case notification data included all persons with new cases of tuberculosis reported to the Brazil national notification system (Sistema de Informacao de Agravos de Notificao; http://portalsinan.saude.gov.br/) between January 1, 2001, and December 31, 2015, and were tabulated by the municipality of residence at the time of case notification, by age, sex and HIV status. HIV incidence was not estimated from case notification because only a subset of HIV cases, persons with AIDS, were notifiable prior to 2014. An annual population series by age and sex for each municipality was obtained from the Brazilian Institute for Geography and Statistics [32].

To inform the model, we included covariates with a known or postulated epidemiologic relationship with HIV or TB infection, progression to active disease, or mortality (Additional file 1: Table S2). Covariates applied annually at the level of the municipality included population density, adjusted monthly income, literacy rate, outdoor air pollution, proportion of population in prison, ambient temperature, household crowding, night-time light brightness, and population-level coverage of Family Health programme teams. Data sources and processing are described in Additional file 1: Table S2. Model comparisons using different covariate sets are described in Additional file 1: Table S3. Additional covariates were estimated annually at the state level from the GBD 2016 study due to lack of available data at the municipality level. These included HIV prevalence, smoking prevalence, diabetes (fasting plasma glucose in mmol/L), alcohol consumption (litres of pure alcohol per capita per year), indoor air pollution prevalence, and a TB risk factor index [33]. However, the addition of these state-level covariates did not substantially alter TB or HIV mortality estimates (Additional file 1: Table S3); they were not included in the final model in an effort to simplify the model.

Municipality boundaries changed to accommodate new municipalities in a small number of cases between 2001 and 2015. Municipalities that had undergone a boundary change during the period of the analysis were merged to create a stable unit. Of the 5565 municipalities present in Brazil in 2015, boundary changes during the analysis period required merging to form 5477 geographic units for analysis. Details of these shifts are provided in Additional file 1: Table S4.

### Cause of death attribution

Standardisation of vital registration data was done based on methods developed in GBD 2016 [34]. In this study, each death was attributed to a single underlying cause that fit within a hierarchy of mutually exclusive and collectively exhaustive causes. The portion of deaths coded with ICD-10 codes that could not be underlying causes of death or were non-specific causes were redistributed according to a framework for processing so-called garbage codes developed by Naghavi et al. [35].

Mortality rates for TB, HIV, and TB among PLHIV were estimated for this study. TB deaths among PLHIV were estimated as a subset of the burden of HIV/AIDS deaths to maintain consistency with GBD and ICD-10 convention [34]. The ICD-10 codes corresponding to each cause estimated here are listed in Additional file 1: Table S1. The patterns of death redistribution through the processing algorithm to each category of TB without HIV and HIV are shown in Additional file 1: Figures S2 and S3, respectively.

### Statistical analysis

Mortality due to TB and HIV (including HIV/TB) was estimated separately by cause and sex using a small area estimation approach developed by Dwyer-Lindgren et al. [36]. This approach applies a Bayesian spatially explicit mixed effects regression model. Conditional autoregressive distributions were used to smooth over age, year and municipality. Covariates for each municipality and year were included as fixed effects (Additional file 1). One thousand draws (i.e. candidate maps) were sampled from the posterior distributions of modelled parameters. Point estimates were produced from the mean of these draws, and uncertainty intervals were generated from the 2.5th to 97.5th percentiles for each age, sex, year, municipality, and cause. Population-weighted municipal-level estimates for each cause and sex were aggregated to the level of the administrative state and Federal District (*n* = 27) for calibration to state-level estimates from GBD 2016 [4]. The posterior probability of a positive or negative association with the outcomes of TB and HIV mortality was estimated for each covariate.

### Model validation

Separate model validation datasets were defined for TB and HIV mortality using municipalities with large numbers of deaths and small year-by-year variation in TB and HIV mortality rates [37]. Further details of statistical comparison between models are provided in the Additional file 1, including Additional file 1: Table S5.

### Case fatality analysis

Case fatality ratios for TB were calculated jointly for persons with and without HIV infection due to unrecorded HIV status in nearly 40% of TB case notification records, with completeness of reporting improving during the period of analysis. Case fatality analysis was restricted to 2001 to 2014 due to an incomplete set of case notification data for 2015. For this analysis, TB mortality events in persons with and without HIV infection were summed for each age, sex, year and municipality. These combined TB and HIV-TB mortality events were modelled using the small area approach described above and calibrated to state-level estimates from GBD 2016. TB incidence in persons with and without HIV infection was modelled from TB case notifications using the same approach and calibrated to state-level TB incidence estimates from GBD 2016. Age-standardised, sex-specific TB and HIV-TB deaths were divided by the age-standardised TB incidence for the corresponding sex, year and municipality. While persons who die from TB may not die in the same year that their case is notified, this is the standard calculation for this TB metric [1].

## Results

### National-level geographic patterns by municipality and notable time trends

**Fig. 1:**
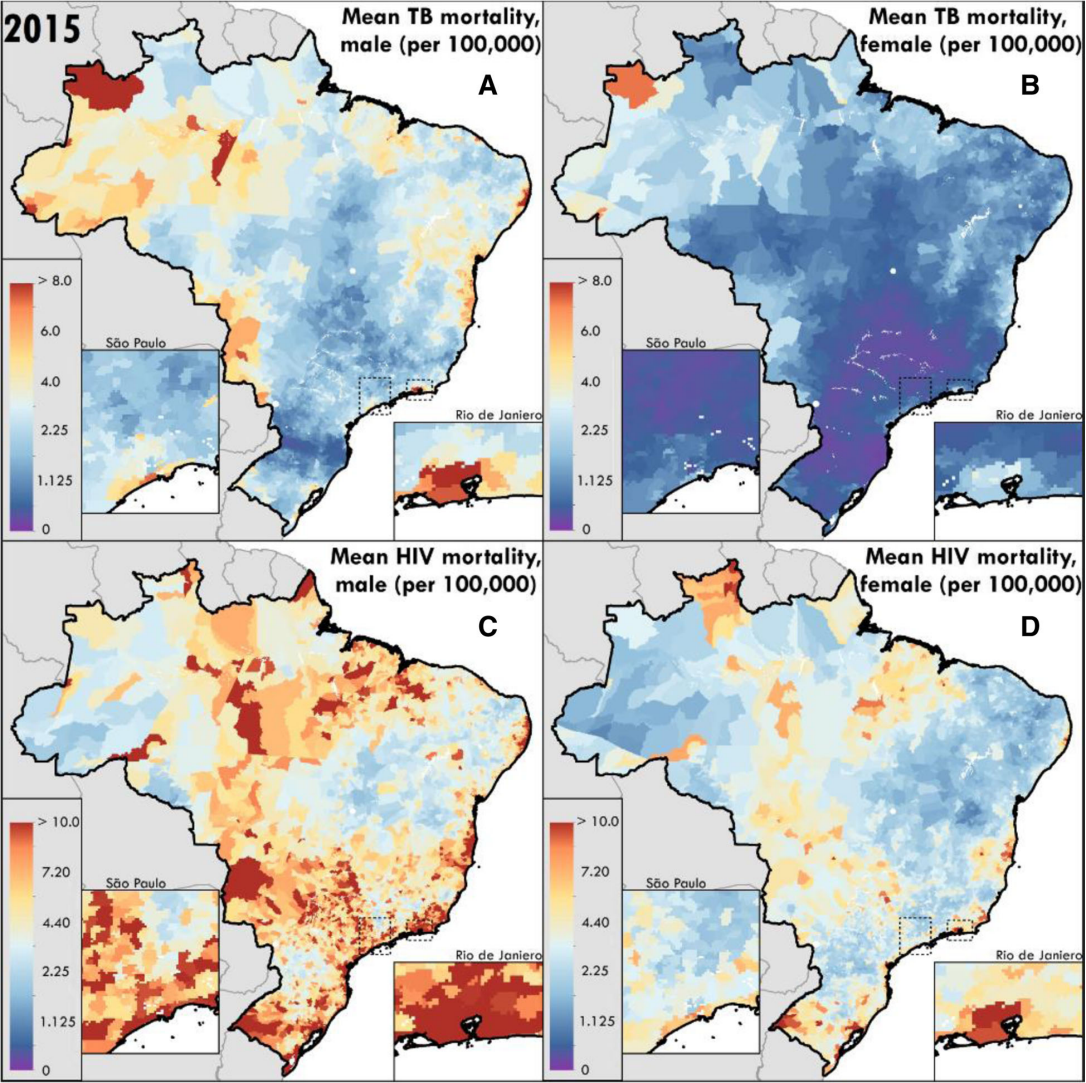
Mean mortality rate per 100,000 population in 2015 for **a** TB among males, **b** TB among females, **c** HIV among males, and **d** HIV among females modelled by municipality in Brazil (*n* = 5477 stable units). All rates are age-standardised and calibrated to the Global Burden of Diseases Study 2016

**Fig. 2:**
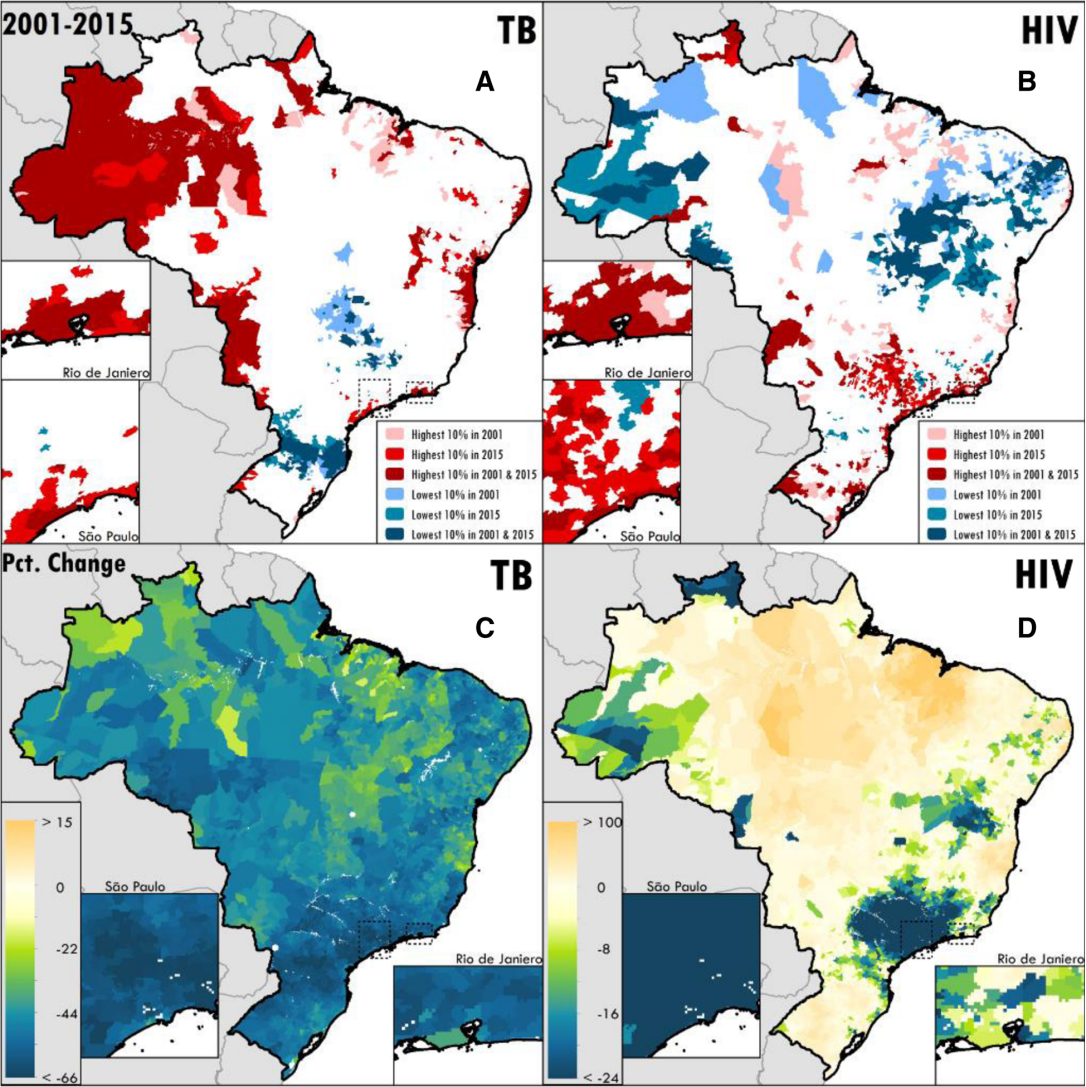
Age-standardised mortality for **a** TB and **b** HIV in highest-burden versus lowest-burden municipalities in select years. Percent change in age-standardised mortality rate between 2001 and 2015 for **c** TB and **d** HIV by municipality in Brazil

TB and HIV mortality rates varied substantially by municipality within the nation during 2001–2015 (Fig. 1). National age-standardised mortality due to TB in persons without HIV decreased by nearly 50% from 6.7 (95% uncertainty interval (UI) 6.5–6.9) deaths per 100,000 in 2001 to 3.5 (95% UI 3.4–3.6) deaths per 100,000 in 2015 among males, and from 2.3 (95% UI 2.2–2.4) deaths per 100,000 in 2001 to 1.2 (95% UI 1.1–1.2) deaths per 100,000 in 2015 among females. National age-standardised mortality due to HIV was 11.0 (95% UI 10.8–11.2) deaths per 100,000 in 2001 versus 8.7 (95% UI 8.5–8.8) deaths per 100,000 in 2015 among males, and 5.0 (95% UI 4.8–5.1) deaths per 100,000 in 2001 versus 4.4 (95% UI 4.2–4.5) deaths in 2015 among females. Despite national-level declines, the majority of municipalities demonstrated increases in HIV mortality during this period, while TB mortality declined in nearly all municipalities (Fig. 2).

**Fig. 3:**
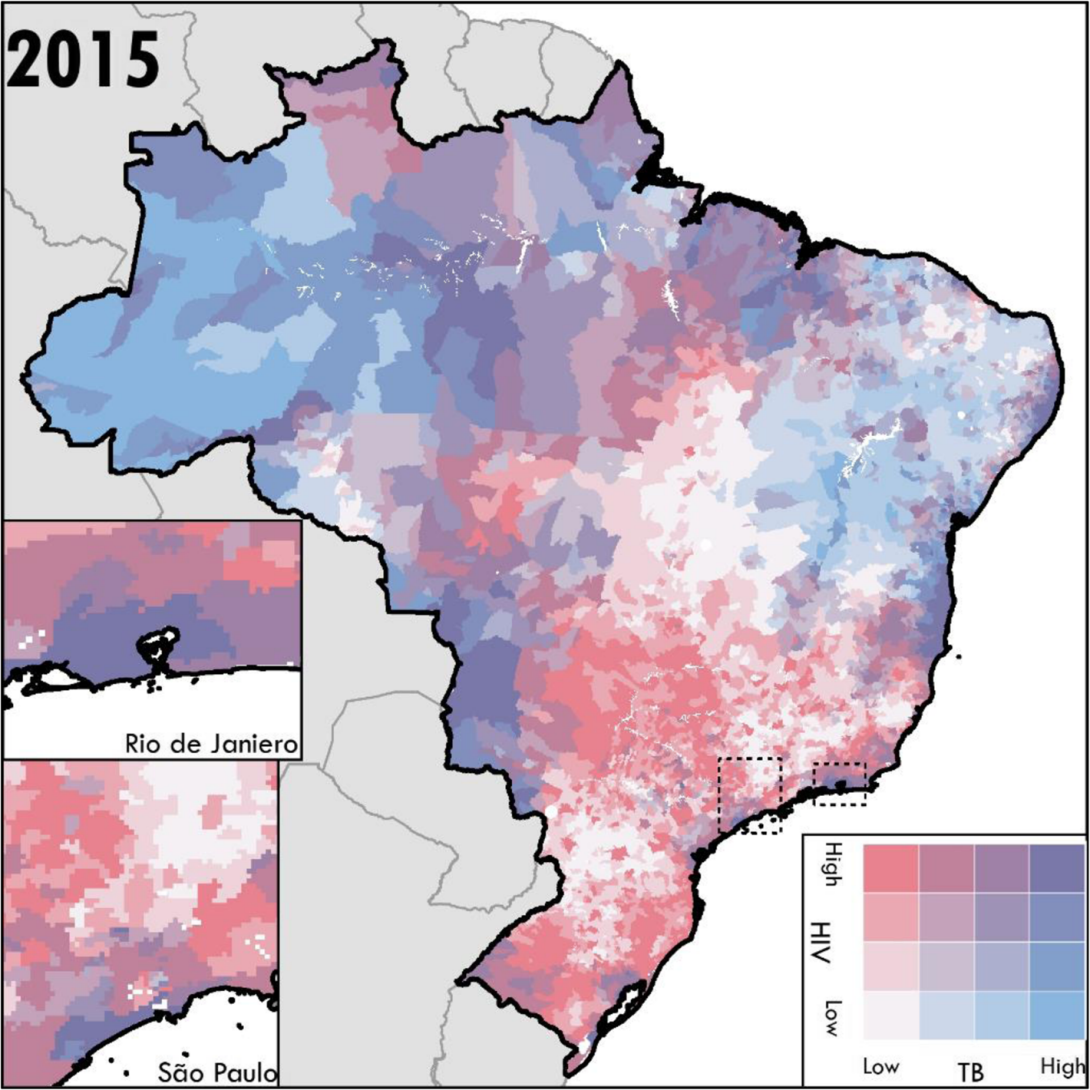
Joint burdens of mortality due to HIV (including TB deaths in people living with HIV) and TB in persons without HIV infection by Brazilian municipality in 2015

The municipalities with mortality rates in the 90th percentile nationally for TB and HIV had mortality rates more than three times higher than those in the 10th percentile nationally (Additional file 1: Table S6). For TB, nearly 70% of municipalities with male mortality rates and more than 75% of municipalities with female mortality rates greater than the 90th percentile in 2001 remained in the 90th percentile in 2015 (Fig. 2, Additional file 1: Table S6). The highest-burden municipalities were less constant for HIV mortality; between 55% and 61% of the municipalities with male or female mortality rates greater than the 90th percentile in 2001 remained in the 90th percentile in 2015 (Fig. 2, Additional file 1: Table S6). TB mortality in persons without HIV and HIV mortality exhibited somewhat different spatial patterns in 2015, with high burdens of TB mortality in persons without HIV infection in the north-western Amazon regions. The joint burdens of TB in persons without HIV infection and HIV mortality were high in the large coastal cities, and the northern states of Amapa and Maranhao (Fig. 3). HIV mortality was also high in inland municipalities in Sao Paulo state, which demonstrated relatively lower TB mortality burden in persons without HIV infection (Fig. 3).

### Mortality variation by municipalities within states

**Fig. 4:**
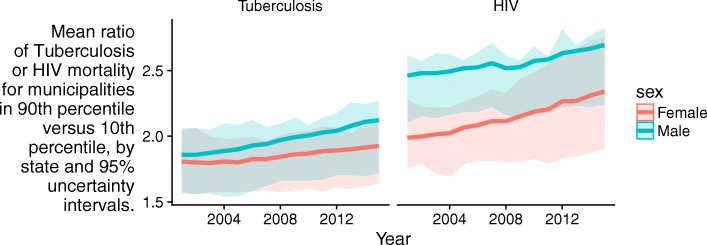
Mean ratio of TB or HIV mortality for municipalities in 90th percentile versus 10th percentile, by state, with 95% uncertainty intervals

TB and HIV mortality varied between municipalities located in the same state (Additional file 1: Figure S4). State-level TB mortality rate ratios for municipalities in the 90th percentile versus those in the 10th percentile varied from 1.4 in Acre to 2.9 in Minas Gerais for males and 1.4 in Rio Grande do Norte to 2.3 in Minas Gerais and Rio de Janeiro for females in 2001. They ranged from 1.6 in Piaui and Goias to 3.3 in Rio de Janiero for males and 1.5 in Amapa, Rio Grande do Norte, and Paraiba to 3.1 in Mato Grosso do Sul for females in 2015. There was an overall trend toward increasing inequality within states over time (Fig. 4a). State-level HIV mortality rate ratios for municipalities in the 90th percentile versus those in the 10th percentile varied from 1.8 in Piaui to 3.4 in Rio Grande do Sul for males and 1.4 in Rio Grande do Norte and Piaui to 3.1 in Santa Catarina for females in 2001. They ranged from 2.0 in Rio Grande do Norte to 4.2 in Pernambuco for males and from 1.4 in Amapa to 3.7 in Pernambuco for females in 2015. There was also increasing inequality within states over time for HIV (Fig. 4b).

### Case fatality ratios for TB in all forms

**Fig. 5:**
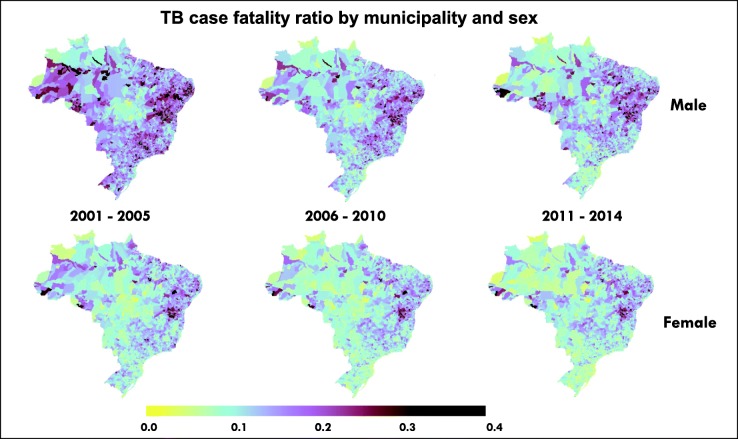
Age-standardised TB all-forms case fatality ratio by municipality and sex. Mapped values are means per year bin. TB all-forms estimation includes persons with and without HIV infection. Estimates are calibrated to the Global Burden of Diseases Study 2016

National TB case fatality ratios, including TB in PLHIV, ranged from 11% to 17% for males and 8% to 11% for females with decreasing values over time (Additional file 1: Figure S7). The proportion of municipalities meeting the WHO End TB Strategy target of a case fatality less than 10%, for males versus females, respectively, was 15% and 40% aggregated over years 2001–2005, 28% and 48% over 2006–2010, and 36% and 54% over 2011–2014. Figure 5 shows the geographic pattern of TB case fatality ratios over this period.

### Relationships with covariates

Greater population coverage of Family Health programme teams was associated with lower TB and HIV mortality (Additional file 1: Table S7). Higher population income was associated with lower TB mortality but higher HIV mortality. Several covariates were associated with higher rates of TB and HIV mortality, including greater household crowding, population density, outdoor air pollution, population literacy, proportion of the male or female population in prison, and higher air temperature.

## Discussion

Despite marked progress nationally in reducing deaths due to TB and concentrated gains for HIV, substantial inequality in TB and HIV burden are apparent at each geographic level of analysis. Trends in within-state variation for TB were driven by faster mortality reductions in the lowest-burden municipalities relative to slower improvement in the highest-burden areas, the majority of which remained in the highest-burden decile at the end of the 15-year interval. HIV mortality declines in highly populated, high-burden areas drove national-level declines, but the majority of municipalities demonstrated an increase in HIV mortality rate during this period, which was also observed in prior studies [38]. Evaluation of the municipalities with the greatest mortality improvements may identify successful strategies that could be extended to areas experiencing increases or slower declines.

Mortality estimates disaggregated by sex revealed differences in TB and HIV burden and geographic distribution. Consistent with known TB and HIV epidemiology, we found a greater burden of TB and HIV mortality in males than in females, but also somewhat different spatial patterns by sex [28, 39]. Incarceration is a known risk factor for TB infection, with prisoners (pessoas privadas de liberdade) in Brazil having an estimated TB notification rate more than 30 times that of the non-incarcerated population [40]. HIV prevalence is also higher in Brazilian inmates than in the non-incarcerated population [41–43]. Men comprise more than 90% of the Brazilian prison population. Municipalities with large prison populations, such as several in Sao Paulo state, stand out in the maps showing results for males as having a higher TB incidence and HIV mortality than neighbouring municipalities. In contrast, municipalities where females were at greatest risk for HIV and TB mortality were concentrated along the national border areas and in the interior Amazon.

National-level case fatality ratios for TB improved over the period of this analysis. However, broader efforts are also needed, as only half of municipalities achieved a WHO End TB Strategy case fatality ratio target of < 10% among females and just over one-third of municipalities achieved it among males in the final period of the analysis between 2011 and 2014. Nearly twice as many municipalities achieved the WHO target for females than for males, indicating a critical need for TB treatment completion strategies that successfully engage men. Underreporting of TB case notifications could bias these estimates downward; however, this effect is likely to be small in later years, when reporting completeness is estimated at more than 90% [1]. There was less apparent geographic patterning for case fatality than for TB or HIV mortality, but the burden of higher case fatality ratios appeared to shift from coastal areas to more inland areas over the analysis period.

While increased funds are required to maintain gains and further improve health and equality, congress approved Constitutional Amendment 95 in December 2016, restricting funds allocated to the health sector and providing no real increase in health funding for the next 20 years [44]. This austerity has also extended to other sectors impacting health and wellbeing, including education and public utilities such as sanitation. These policies could stall the important progress made in Brazil over the period of this study.

This work extends previous efforts to model subnational TB and HIV burden by generating estimates that are both nationally comprehensive and fine-scale. It supports calls to collect and analyse TB and HIV data with high spatial resolution in order to inform interventions that are most appropriate to the transmission dynamics in particular settings [45]. Knowledge of the local variation in TB and HIV burden can inform programmatic interventions to improve health outcomes [16]. TB interventions, such as active case finding and mobile testing units, can be resource-intensive and are utilised most effectively when prioritised to high-burden areas [46]. Subnational differences in HIV burden have also been used to develop locally tailored strategies for HIV prevention and elimination [25, 47–49]. However, the benefits of highly geographically resolved disease burden estimation should be weighed against the risk of potentially identifying individuals if analyses of exceptionally rare outcomes are carried out over very small areas.

### Limitations

There are several limitations to this analysis. While adult mortality data in Brazil are assessed to be complete for the period of this analysis, child mortality data are estimated to be < 95% complete in the vital registration system [50]. Other national-level analyses have included additional data sources at a different spatial resolution such as household surveys [51]. Due to the complexity of integrating different data types, only vital registration data were included in this analysis. However, calibrating these estimates to GBD, which includes survey data in all-cause mortality estimation, reduces the undercounting of deaths. Deaths in children under the age of 15 constitute a small proportion of TB (1.6%) and HIV (7%) deaths in Brazil during this period, so the spatial effect of this difference in data sources is not expected to be large. Similarly, TB cases may be under-ascertained in the case notification system. While the overall completeness of TB case notification is estimated currently to be greater than 90%, completeness of reporting may vary spatially [1]. Future work may assess whether factors such as treatment-seeking behaviour and reporting completeness can be used to improve modelling of TB incidence from case notifications.

HIV and TB are under-ascertained as causes of death, and TB is under-ascertained as a contributing cause of death among persons with HIV infection [52]. The GBD mortality redistribution method attempts to correct for these biases. Other correction methods include linkage analysis of HIV and TB surveillance systems [27], or linkage of diagnoses made at health facility encounters with information recorded on death certificates. These could be pursued as additional methods to improve ascertainment of TB and HIV deaths.

### Future directions

There are several additional future directions for this work. First, while the causes of TB mortality and TB incidence are further broken down in GBD analyses into drug-susceptible TB, multidrug-resistant TB and extensively drug-resistant TB, data were not available at the geographic scale of this study to inform analysis by drug resistance categories. Additional geographic detail in data sources would facilitate analysis by drug resistance categories. Second, climatologic variables were included in the TB models as an exploratory analysis due to postulated relationships between air temperature, wind speed and TB transmission [22, 53]. Relationships with these factors may be tested in future spatial models in order to potentially improve estimation of TB burden in areas with minimal health surveillance data. Third, a similar small area estimation approach could be used to estimate all-cause and cause-specific mortality due to other causes at the municipal level in Brazil. Finally, this small area estimation approach to spatial mapping of HIV and TB mortality could be extended to other nations with well-functioning vital registration systems.

## Conclusion

Mortality due to TB and HIV exhibited nearly as much relative variation within Brazilian states as within the nation as a whole. This demonstrates the role for increasing geographic detail in burden estimation to guide precision public health responses. Fewer than half of municipalities met the WHO End TB Strategy target for a case fatality rate of < 10%, indicating priority areas for improvement in order to achieve international targets and improve health equity.

## Additional file

Additional file 1: Data sources, model equations and validation, and additional tables and figures. (DOCX 1360 kb)

## Abbreviations

GBD: Global Burden of Diseases
HIV: human immunodeficiency virus
ICD: International Statistical Classification of Diseases
PLHIV: people living with HIV
TB: tuberculosis
UI: uncertainty interval
WHO: World Health Organization
