# Supplementary material for: Progress toward eliminating TB and HIV deaths in Brazil, 2001–2015: a spatial assessment
Source: BMC Med. 2018 Sep 6;16:144. doi: 10.1186/s12916-018-1131-6 (PMC6125942; doi:10.1186/s12916-018-1131-6)
Supplement: Supplementary file 1 — Data sources, model equations and validation, and additional tables and figures. (DOCX 1360 kb) [file 12916_2018_1131_MOESM1_ESM.docx]

Supplementary Information

Contents

[1.0 Data Sources 2](#_Toc516066485)

[1.1 ICD-10 codes corresponding to each mortality cause 2](#_Toc516066486)

[1.2 Mortality and notifications outcome data citations 2](#_Toc516066487)

[1.3 Covariate data sources 3](#_Toc516066488)

[1.4 Model comparison using different sets of covariates 4](#_Toc516066489)

[1.5 Model geographies 5](#_Toc516066490)

[2.0 Supplementary Methods 5](#_Toc516066491)

[2.1 Redistribution of deaths 5](#_Toc516066492)

[2.2 Formation of stable geographic units 8](#_Toc516066493)

[2.3 Small area estimation model description 10](#_Toc516066494)

[3.0 Model validation 11](#_Toc516066495)

[4.0 Supplementary Results 12](#_Toc516066496)

[4.1 TB and HIV mortality rates, 90^th^ and 10^th^ percentiles 12](#_Toc516066497)

[4.2 Mortality relationships with covariates 13](#_Toc516066498)

[4.3 TB all forms (with and without HIV) mortality, incidence, and case fatality 13](#_Toc516066499)

[5.0 Supplementary References 16](#_Toc516066500)

# 1.0 Data Sources

## ICD-10 codes corresponding to each mortality cause

Table S1: ICD-10 codes corresponding to each cause

| **Cause** | **ICD-10 Codes** |
| --- | --- |
| Tuberculosis | A10 - A19, B90, K67.3, K93.0, M49.0, P37.0, U84.3 |
| HIV/AIDS | B20 - B24 |
| - *HIV/AIDS - Tuberculosis* | B20.0 |

## 1.2 Mortality and notifications outcome data citations

TB and HIV mortality and TB case notification data are cited in the Global Health Data Exchange (ghdx.healthdata.org) and are catalogued independently by year.

Example mortality data citation:

ghdx.healthdata.org/record/brazil-mortality-information-system-deaths-2001

Example case notification data citation:

http://ghdx.healthdata.org/record/brazil-information-system-notifiable-diseases-2001

## 1.3 Covariate data sources

Table S2: Covariates and data sources

| **Covariate** | **Spatial resolution** | **Temporal resolution** | **Source** | **Reference** |
| --- | --- | --- | --- | --- |
| Ambient Air Pollution | Municipality | Annual | Cornell University | Shaddick et al. Data Integration Model for Air Quality: A Hierarchical Approach to the Global Estimation of Exposures to Ambient Air Pollution. *Stat AP*. (2016) |
| Average daily mean temperature | Municipality | Annual | CRUTS | Harris, I., Jones, P. d., Osborn, T. j. & Lister, D. h. Updated high-resolution grids of monthly climatic observations – the CRU TS3.10 dataset. *Int. J. Climatol*. **34**, 623–642 (2014).  University of East Anglia. Climatic Research Unit TS v. 3.24 dataset. Available at: https://crudata.uea.ac.uk/cru/data/hrg/cru_ts_3.24.01/. (Accessed: 24th July 2017). |
| Population density | Municipality | Annual | Brazil Ministry of Health (derived) | Brazil Ministry of Health. DataSUS Tabnet web portal. Available at: http://tabnet.datasus.gov.br/cgi/deftohtm.exe?ibge/cnv/popbr.def. (Accessed: 7 June 2017) |
| Nighttime lights | Municipality | Annual | NOAA DMSP | Savory et al. Intercalibration and Gaussian Process Modeling of Nighttime Lights Imagery for Measuring Urbanization Trends in Africa 2000– 2013. Remote Sens. 9, (2017). |
| Proportion of Population Incarcerated | Municipality | Annual | National | National Council of Justice (CNJ). National Registry of Inspections in Penal Establishments (CNIEP). Available at: http://www.cnj.jus.br/inspecao_penal/mapa.php. (Accessed: 14^th^ August 2017)  Brazil Ministry of Health. DataSUS Tabnet web portal. Available at: http://tabnet.datasus.gov.br/cgi/deftohtm.exe?ibge/cnv/popbr.def. (Accessed: 7 June 2017) |
| Adjusted monthly income | Municipality | 2000, 2010 (interpolated) | IPUMS (derived) | Minnesota Population Center. *Integrated Public Use Microdata Series, International: Version 6.5* [dataset]. Minneapolis: University of Minnesota, 2017. http://doi.org/10.18128/D020.V6.5.  Brazilian Institute of Geography and Statistics (IBGE). Brasilia, Brazil. |
| Literacy rate | Municipality | 2000, 2010 (interpolated) | IPUMS (derived) |  |
| Household crowding | Municipality | 2000, 2010 (interpolated) | IPUMS (derived) |  |
| Coverage of family health teams | Municipality | Annual | Brazil Ministry of Health | Brazil Ministry of Health. Department of Basic Attention. Available at: <http://dab.saude.gov.br/portaldab/historico_cobertura_sf.php>. (Accessed: 21^st^ December 2017) |

Covariates initially present in raster format were aggregated to the municipal level using the mean value weighted by a population raster [1]. Linear interpolation was performed for values between census 2000 and census 2010 and linear trends continued for years 2011-2015.

## 1.4 Model comparison using different sets of covariates

Table S3

| Outcome | Comparison | CI overlap | Mean relative difference in point estimates | Correlation |
| --- | --- | --- | --- | --- |
| TB mortality | Municipal-level covariates only or municipal and state-level covariates | all | 0.0076 | 0.99903 |
| HIV mortality | Municipal-level covariates only or municipal and state-level covariates | all | <0.001 | 0.99968 |
| TB mortality | Prison covariate measured as proportion of population incarcerated versus presence/absence of a prison | all | 0.038 | 0.99931 |
| HIV mortality | Prison covariate measured as proportion of population incarcerated versus presence/absence of a prison | all | 0.151 | 0.99697 |
| TB mortality | Municipal level covariates including population density versus municipal-level covariates with household crowding | all | -0.0053 | 0.99757 |
| HIV mortality | Municipal level covariates including population density versus municipal-level covariates with household crowding | all | 0.0174 | 0.99762 |

## 1.5 Model geographies

Fig S1: Map of Brazilian states (n=26) and Federal District (Distrito Federal)[2]

_
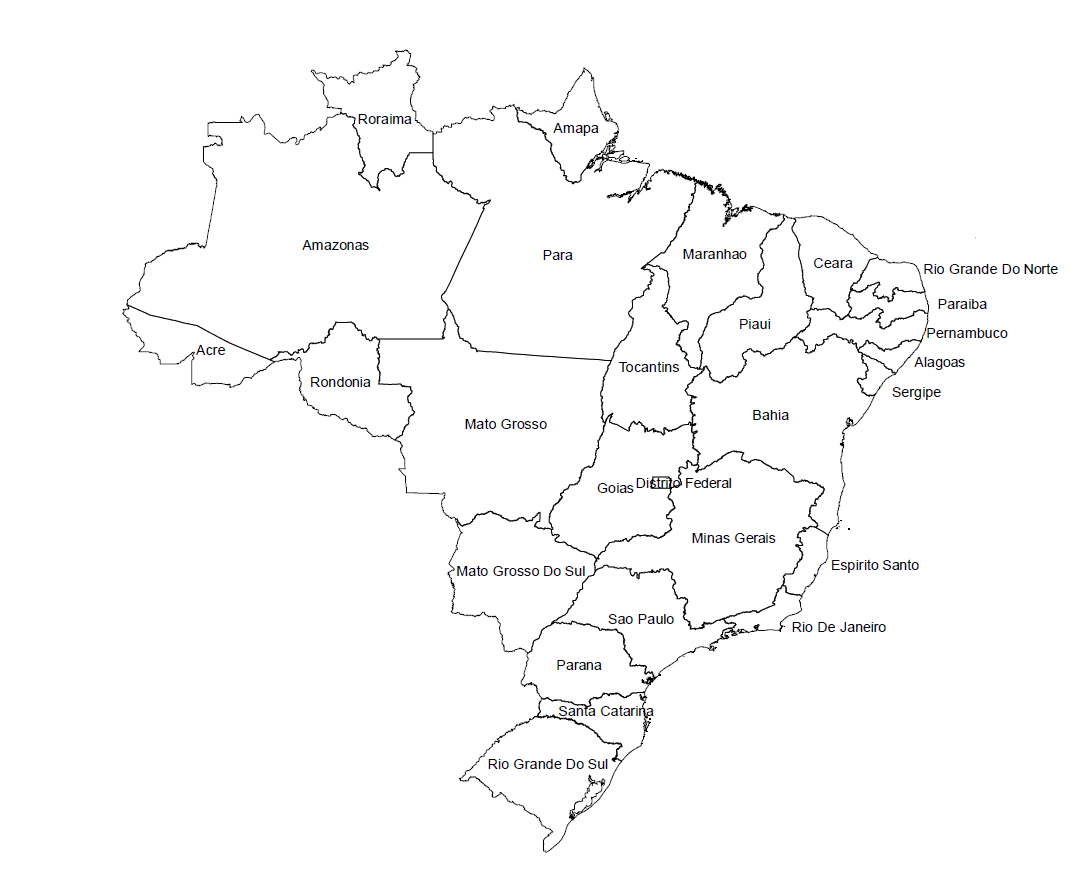
_

# 2.0 Supplementary Methods

##

## Analysis code will be made available upon request.

## 2.1 Redistribution of deaths

Deaths were redistributed to the causes of Tuberculosis and HIV/AIDS according to the methods of the Global Burden of Diseases Study 2016 [3]. A defining feature of this process is the re-assignment of deaths originally assigned to so-called “garbage codes”, which are ICD codes that that cannot be underlying causes of death, are intermediate causes of death rather than the underlying cause, or lack specificity in coding. The diagrams below show the proportion of deaths attributed to tuberculosis (Fig. S2) and HIV/AIDS (Fig. S3) which were redistributed from other causes.

Fig. S2: Redistribution diagram for deaths due to tuberculosis in persons without HIV infection. Categories on the left-hand side show the contribution of deaths within various ICD-10 codings to the final analysis dataset, represented on the right-hand side. The “Original” category on the left-hand side represents deaths already coded to tuberculosis before redistribution.


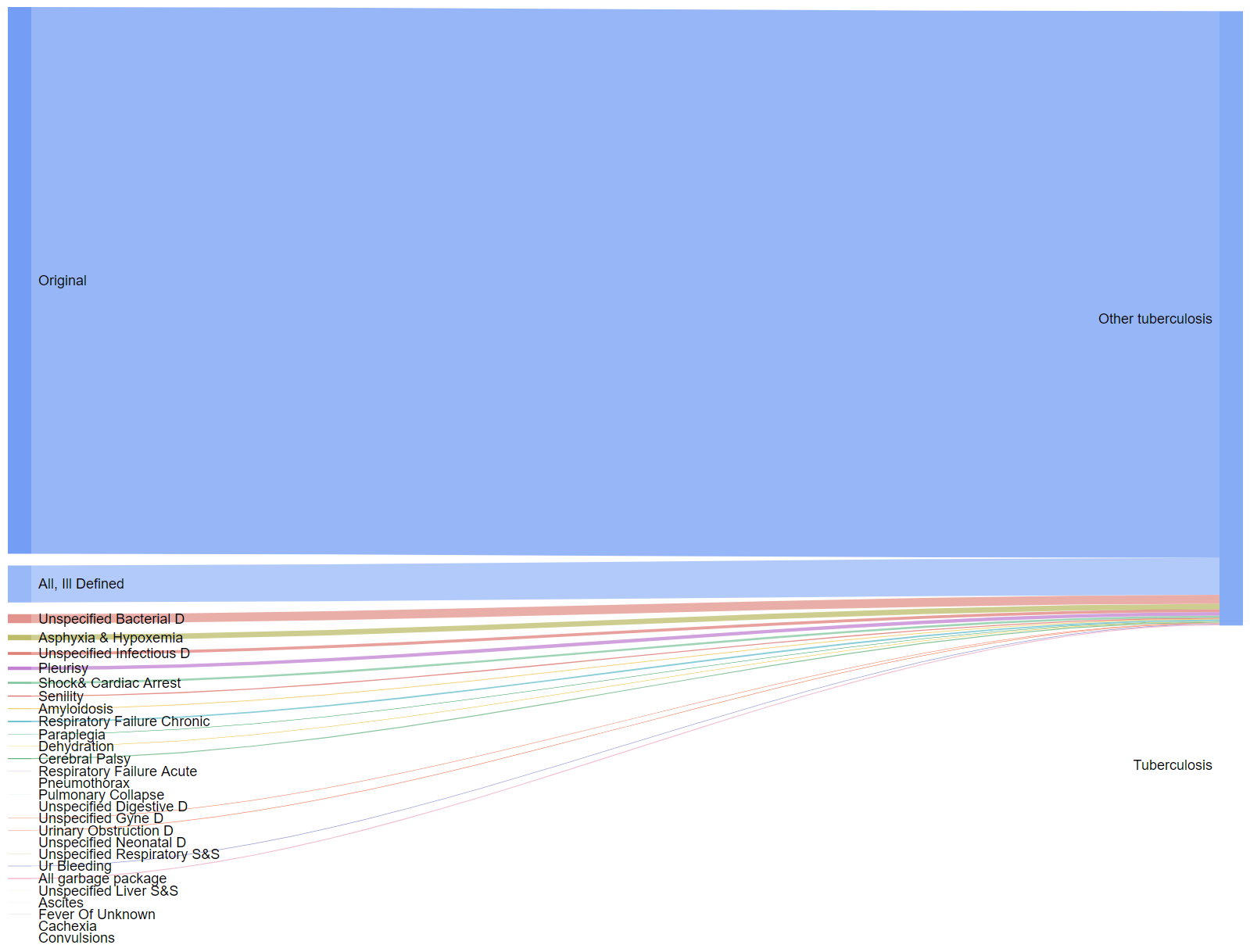


Fig. S3: Redistribution diagram for deaths due to HIV/AIDS. Categories on the left-hand side show the contribution of deaths coded ICD-10 codings to the final analysis dataset, represented on the right-hand side. The “Original” category on the left-hand side represents deaths already coded to HIV/AIDS before redistribution.
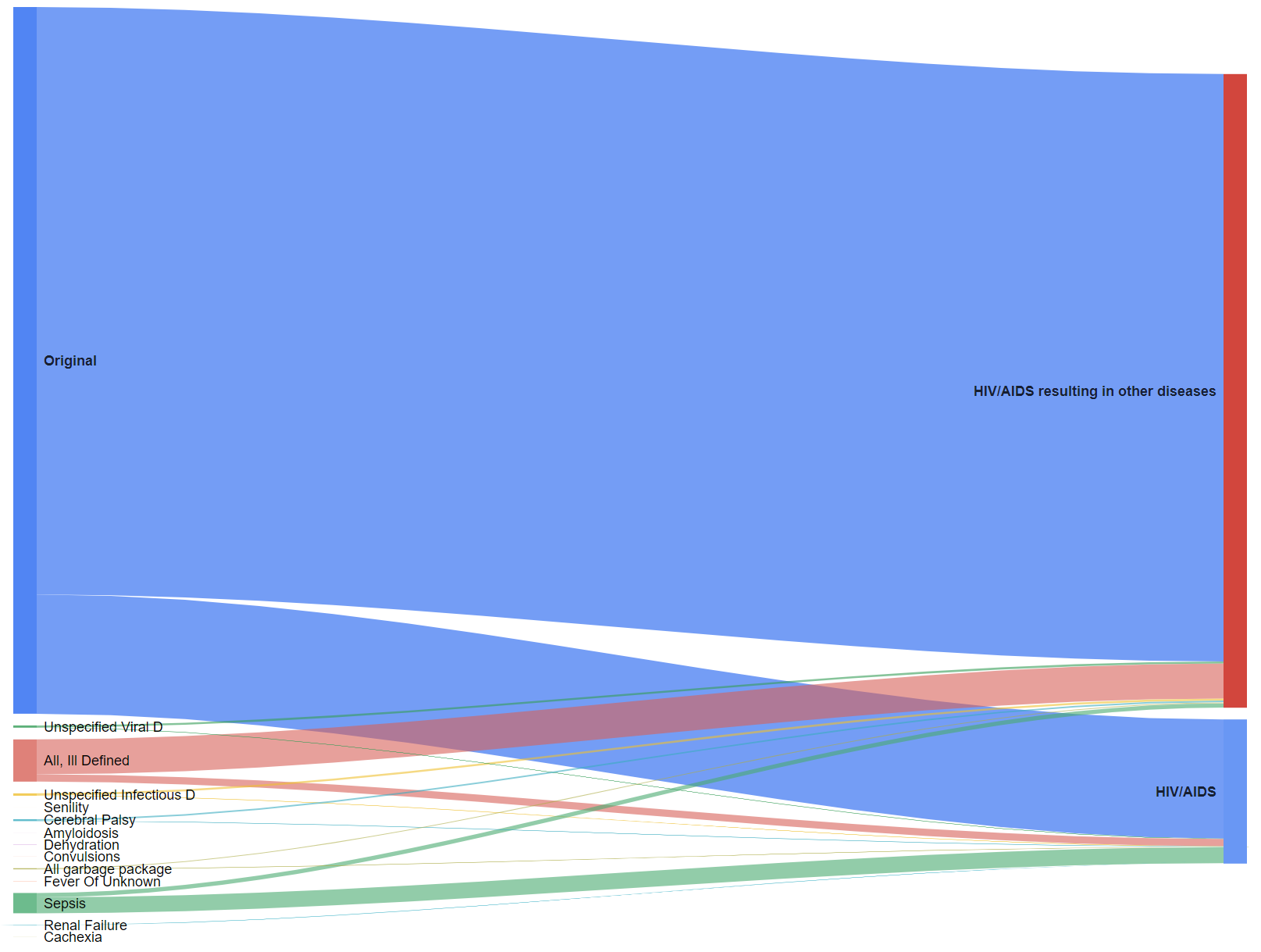


## 2.2 Formation of stable geographic units

Boundary changes required merging a small number of municipalities to create stable geographic units for analysis. Details of these merges are given in Table S4.

Table S4: Municipalities merged to form stable geographic units of analysis

| **State** | **Group** | **Areas** |
| --- | --- | --- |
| Alagoas | 1 | Coruripe (270230), Jequia da Praia (270375), Sao Miguel dos Campos (270860) |
| Bahia | 1 | Barreiras (290320), Luis Eduardo Magalhaes (291955) |
| Bahia | 2 | Barrocas (290327), Serrinha (293050) |
| Espirito Santo | 1 | Colatina (320150), Governador Lindenberg (320225) |
| Goias | 1 | Anapolis (520110), Campo Limpo de Goias (520485) |
| Goias | 2 | Ceres (520540), Ipiranga de Goias (521015) |
| Goias | 3 | Gameleira de Goias (520815), Silvania (522060) |
| Goias | 4 | Itaja (521080), Lagoa Santa (521225) |
| Mato Grosso | 1 | Agua Boa (510020), Nova Nazare (510617) |
| Mato Grosso | 2 | Alto Boa Vista (510035), Bom Jesus do Araguaia (510185), Cocalinho (510310), Novo Santo Antonio (510631), Ribeirao Cascalheira (510718), Sao Felix do Araguaia (510785), Serra Nova Dourada (510788) |
| Mato Grosso | 3 | Aripuana (510140), Colniza (510325), Rondolandia (510757) |
| Mato Grosso | 4 | Caceres (510250), Curvelandia (510343), Lambari d'Oeste (510523), Mirassol d'Oeste (510562) |
| Mato Grosso | 5 | Claudia (510305), Itauba (510455), Nova Santa Helena (510619) |
| Mato Grosso | 6 | Conquista d'Oeste (510336), Pontes e Lacerda (510675), Vale de Sao Domingos (510835) |
| Mato Grosso | 7 | Ipiranga do Norte (510452), Itanhanga (510454), Tapurah (510800) |
| Mato Grosso | 8 | Nova Mutum (510622), Santa Rita do Trivelato (510776) |
| Mato Grosso | 9 | Novo Sao Joaquim (510628), Santo Antonio do Leste (510779) |
| Mato Grosso | 10 | Santa Cruz do Xingu (510774), Sao Jose do Xingu (510735) |
| Mato Grosso do Sul | 1 | Agua Clara (500020), Camapua (500260), Chapadao do Sul (500295), Costa Rica (500325), Figueirao (500390), Paraiso das Aguas (500627) |
| Para | 1 | Mojui dos Campos (150475), Santarem (150680) |
| Piaui | 1 | Altos (220040), Pau d'Arco do Piaui (220779) |
| Piaui | 2 | Aroeiras do Itaim (220095), Picos (220800) |
| Piaui | 3 | Nazaria (220672), Teresina (221100) |
| Rio Grande do Norte | 1 | Jundia (240615), Varzea (241470) |
| Rio Grande do Sul | 1 | Acegua (430003), Bage (430160) |
| Rio Grande do Sul | 2 | Agua Santa (430005), Caseiros (430495), Ibiaca (430980), Santa Cecilia do Sul (431673), Tapejara (432090) |
| Rio Grande do Sul | 3 | Almirante Tamandare do Sul (430047), Carazinho (430470) |
| Rio Grande do Sul | 4 | Arroio do Padre (430107), Pelotas (431440) |
| Rio Grande do Sul | 5 | Augusto Pestana (430150), Boa Vista do Cadeado (430222), Boa Vista do Incra (430223), Bozano (430258), Cruz Alta (430610), Fortaleza dos Valos (430845), Ijui (431020) |
| Rio Grande do Sul | 6 | Barao de Cotegipe (430170), Erechim (430700), Jacutinga (431090), Paulo Bento (431413), Ponte Preta (431478), Quatro Irmaos (431531) |
| Rio Grande do Sul | 7 | Bento Goncalves (430210), Pinto Bandeira (431453), Pinto Bandeira (431454) |
| Rio Grande do Sul | 8 | Caibate (430330), Mato Queimado (431217) |
| Rio Grande do Sul | 9 | Campinas do Sul (430380), Cruzaltense (430613) |
| Rio Grande do Sul | 10 | Canudos do Vale (430461), Forquetinha (430843), Lajeado (431140), Progresso (431515) |
| Rio Grande do Sul | 11 | Capao Bonito do Sul (430462), Lagoa Vermelha (431130) |
| Rio Grande do Sul | 12 | Capao do Cipo (430465), Santiago (431740), Sao Miguel das Missoes (431915) |
| Rio Grande do Sul | 13 | Constantina (430580), Novo Xingu (431346) |
| Rio Grande do Sul | 14 | Coqueiro Baixo (430583), Nova Brescia (431300), Relvado (431545) |
| Rio Grande do Sul | 15 | Coronel Pilar (430593), Garibaldi (430860), Roca Sales (431580) |
| Rio Grande do Sul | 16 | Ernestina (430705), Ibirapuita (430995), Tio Hugo (432146), Victor Graeff (432320) |
| Rio Grande do Sul | 17 | Esmeralda (430740), Pinhal da Serra (431446) |
| Rio Grande do Sul | 18 | Espumoso (430750), Jacuizinho (431087), Salto do Jacui (431645) |
| Rio Grande do Sul | 19 | Herval (430710), Pedras Altas (431417), Pinheiro Machado (431450) |
| Rio Grande do Sul | 20 | Imigrante (431036), Teutonia (432145), Westfalia (432377) |
| Rio Grande do Sul | 21 | Itati (431065), Terra de Areia (432143) |
| Rio Grande do Sul | 22 | Lagoa Bonita do Sul (431123), Sobradinho (432070) |
| Rio Grande do Sul | 23 | Marata (431179), Montenegro (431240), Salvador do Sul (431650), Sao Jose do Sul (431861) |
| Rio Grande do Sul | 24 | Palmeira das Missoes (431370), Sao Pedro das Missoes (431936) |
| Rio Grande do Sul | 25 | Rolador (431595), Sao Luiz Gonzaga (431890) |
| Rio Grande do Sul | 26 | Santa Margarida do Sul (431697), Sao Gabriel (431830) |
| Rio de Janeiro | 1 | Mesquita (330285), Nova Iguacu (330350) |
| Santa Catarina | 1 | Balneario Rincao (422000), Icara (420700) |
| Santa Catarina | 2 | Laguna (420940), Pescaria Brava (421265) |

## 2.3 Small area estimation model description

The following small area model was estimated separately for males and females:

*E_j,t,a_* ~ Poisson (*m_j,t,a_* · *P_j,t,a_*)

log(*m_j,t,a_*) = *β_0_* + *β_1_* · *X_j,t_* + *γ_1,a,t_* + *γ_2,j_* + *γ_3,j_* ·*t* + *γ_4,j,t_*

where

- *j, t*, and *a* are indices for municipality, calendar year (2001 – 2015), and age group (0-4, 5-9, …, 75-79, and 80+), respectively;
- *E_j,t,a_* and *P_j,t,a_* are the number of events (eg cause-specific deaths or case notifications) and the population count, respectively, in municipality *j*, year *t*, and age group *a*;
- *m_j,t,a_* is the underlying cause-specific event rate in municipality *j*, year *t*, and age group *a;*
- *β_0_* is an intercept;
- *X_j,t_* is a vector of covariates for municipality *j* and year *t*, and *β_1_* is the associated vector of regression coefficients;
- *γ_1,a,t_* is an age group- and year-level random intercept;
- *γ_2,j_* is a municipality-level random intercept;
- *γ_3,j_* is a municipality-level random slope on year;
- and *γ_4,j,t_* is a municipality- and year-level random intercept.

*γ_2_* and *γ_3_* were each assumed to follow a conditional autoregressive distribution where the full conditional distribution is given by:

$\gamma_{j}|\gamma_{k\sim j}, \sigma^{2}, \rho\sim Normal\left( \frac{\rho\cdot\sum_{k \sim j} \gamma_{k}}{n_{j} \cdot\rho+ 1 - \rho}, \frac{\sigma^{2}}{n_{j} \cdot\rho+ 1 - \rho} \right)$

where

- *k ~ j* indicates the set of municipalities *k* that are adjacent to municipality *j*;
- *n_j_* is the number of counties in *k ~ j*;
- and σ^2^ and $\rho$ are variance and correlation parameters, respectively.

Variance (σ^2^) and correlation ($\rho)$ parameters were fitted from the model, and indicate the degree of spatial variation and spatial smoothness, respectively. $\rho$ ranged 0 to 1 with higher values indicating greater spatial smoothness. *γ_1_* was assumed to follow a conditional autoregressive distribution with adjacent years and age groups (rather than municipalities) informing the neighboring structure. *γ_4_* was assumed to follow an identical and independently distributed Normal distribution. The model was fit using the Template Model Builder[4] package in R version 3.2.4 [5].

# 3.0 Model validation

Model coverage and correlation were evaluated separately for TB and HIV mortality using a variation of the method developed by Srebotnjak and colleagues [6] and adapted by Dwyer-Lindgren and colleagues [7]. For this analysis, a gold standard set of municipalities was defined separately for TB and HIV mortality based on municipalities with large numbers of TB or HIV deaths and small year-by-year variation in mortality rate. The population of these municipalities was sampled down to age- and sex- representative smaller populations of 1000, 2000, 3000, 5000, 12000, and 80000 (representing the 1^st^, 5^th^, 10^th^, 25^th^, 50^th^, and 80^th^ percentiles of 2015 populations of Brazilian municipalities) to examine the performance of the model with smaller sets of events. Deaths were sampled from a Poisson distribution, and modelled with ten iterations at each population size. Model estimates were compared to the mortality rates from the gold standard set of municipalities for calculation of the metrics below.

Table S5: Validation metrics for age-standardised TB and HIV mortality rates derived from SAE model, by population size

| Infection | Population Size | Mean relative error | Root mean squared error | Coverage |
| --- | --- | --- | --- | --- |
| TB | 1000 | -0.002 | 1.813 | 0.835 |
|  | 2000 | 0.003 | 1.817 | 0.831 |
|  | 3000 | 0.011 | 1.802 | 0.838 |
|  | 5000 | 0.015 | 1.800 | 0.830 |
|  | 12000 | 0.032 | 1.784 | 0.825 |
|  | 80000 | 0.047 | 1.300 | 0.782 |
| HIV | 1000 | 0.031 | 4.738 | 0.853 |
|  | 2000 | 0.038 | 4.577 | 0.851 |
|  | 3000 | 0.036 | 4.428 | 0.853 |
|  | 5000 | 0.034 | 4.107 | 0.855 |
|  | 12000 | 0.041 | 3.458 | 0.849 |
|  | 80000 | 0.040 | 2.251 | 0.830 |

# 4.0 Supplementary Results

## 4.1 TB and HIV mortality rates, 90^th^ and 10^th^ percentiles

Table S6: TB and HIV mortality rate in 90^th^ percentile versus 10^th^ percentile of municipalities nationally by sex

| Infection | Sex | %tile | Mortality rate in  2001  (95% UI) | Mortality rate in  2015  (95% UI) | Proportion of municipalities present in both years |
| --- | --- | --- | --- | --- | --- |
| TB (no HIV) | Male | 90 | 7.01 (4.59 – 10.0) | 4.14 (2.39 – 6.82) | 383/548 (69.9%) |
|  |  | 10 | 2.35 (1.34 – 3.77) | 1.11 (0.54 – 1.96) | 373/548 (68.1%) |
| TB (no HIV) | Female | 90 | 3.10 (1.96 – 4.72) | 1.60 (0.89 – 2.65) | 412/548 (75.2%) |
|  |  | 10 | 0.86 (0.49 – 1.37) | 0.41 (0.20 – 0.71) | 415/548 (75.7%) |
| HIV | Male | 90 | 10.3 (6.67 – 15.7) | 9.37 (5.81 – 15.0) | 333/548 (60.8%) |
|  |  | 10 | 2.52 (1.30 - 4.39) | 2.82 (1.25 – 5.23) | 318/548 (58.0%) |
| HIV | Female | 90 | 5.32 (3.28 – 8.22) | 4.94 (2.74 – 8.56) | 303/548 (55.3%) |
|  |  | 10 | 1.81 (0.92 – 3.09) | 1.67 (0.74 – 3.14) | 324/548 (59.1%) |

Fig S4: TB and HIV mortality rates in 90^th^ percentile versus 10^th^ percentile of municipalities by state plotted across years, both sexes


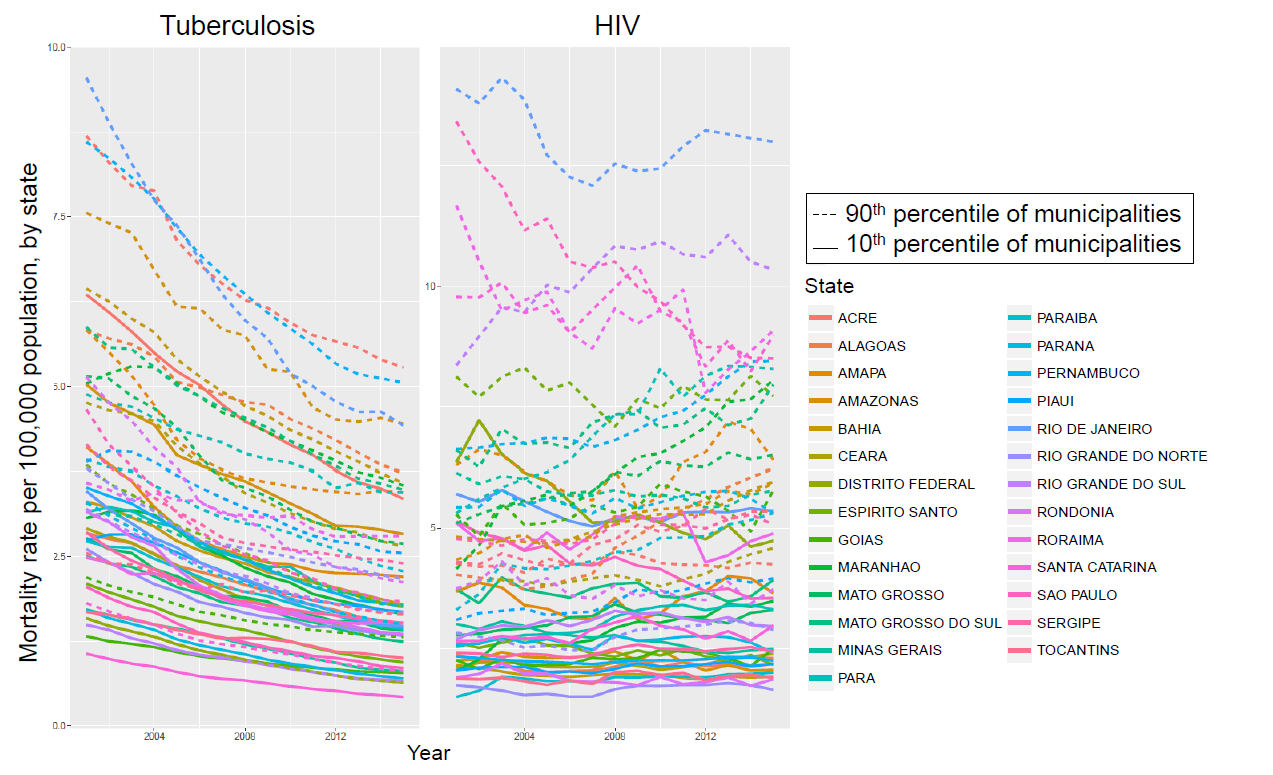


## 4.2 Mortality relationships with covariates

The posterior probability of a given covariate having a positive relationship with the outcomes of TB and HIV mortality was estimated as the proportion of draws (out of 1000) in which the coefficient was positive. Values near 1 indicate a high degree of confidence that the relationship between a covariate and the outcome is positive, while values near zero indicate a high degree of confidence that the relationship between a covariate the outcome is negative. Intermediate values closer to 0.5 indicate less certainty about the direction of the relationship between a given covariate and the outcome.

Table S7: Proportion of draws in which each covariate has a positive relationship with mortality outcome (*ie*, coefficient >0).

| **Covariate** | **Male TB** | **Female TB** | **Male HIV** | **Female HIV** |
| --- | --- | --- | --- | --- |
| Household crowding | >0.999 | >0.999 | >0.999 | >0.999 |
| Population density | 0.996 | 0.934 | >0.999 | >0.999 |
| Female prison population | 0.650 | 0.719 | >0.999 | >0.999 |
| Male prison population | >0.999 | 0.906 | >0.999 | >0.999 |
| Ambient air temperature | >0.999 | >0.999 | 0.662 | 0.258 |
| Nighttime lights | 0.545 | 0.979 | >0.999 | 0.955 |
| Outdoor air pollution | >0.999 | 0.899 | >0.999 | >0.999 |
| Literacy rate | >0.999 | 0.993 | >0.999 | 0.996 |
| Monthly income | 0.030 | 0.054 | >0.999 | >0.999 |
| Family health program coverage | 0.048 | 0.086 | 0.017 | 0.120 |
| Covariates are estimated at the level of the municipality from sources detailed in Table S2. | | | | |

## 4.3 TB all forms (with and without HIV) mortality, incidence, and case fatality

Deaths attributed to TB in people with and without HIV were summed by age group, sex, municipality, and year and then modelled using the small area estimation model (Fig S5). These model results are the numerator for the case fatality ratio calculation. TB case notifications in persons with or without HIV infection were modelled to estimate incidence using the small area estimation approach (Fig S6). These results are the denominator for the case fatality ratio calculation.

Fig S5: Age-standardised TB all-forms (includes TB in PLHIV) mortality per 100,000 population by sex in select years


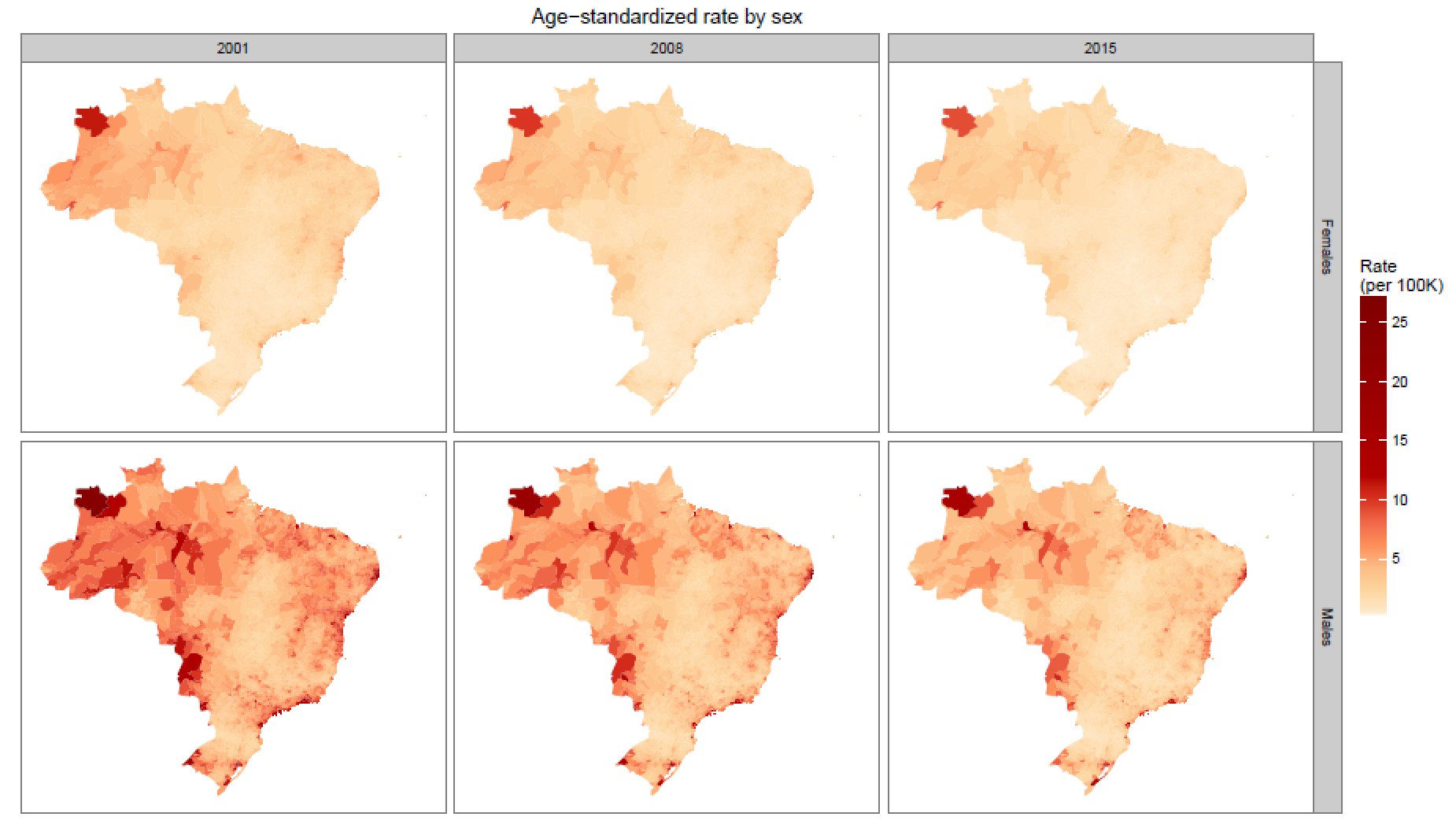


Fig S6: Age-standardised TB all-forms (includes TB in PLHIV) incidence per 100,000 population by sex in select years


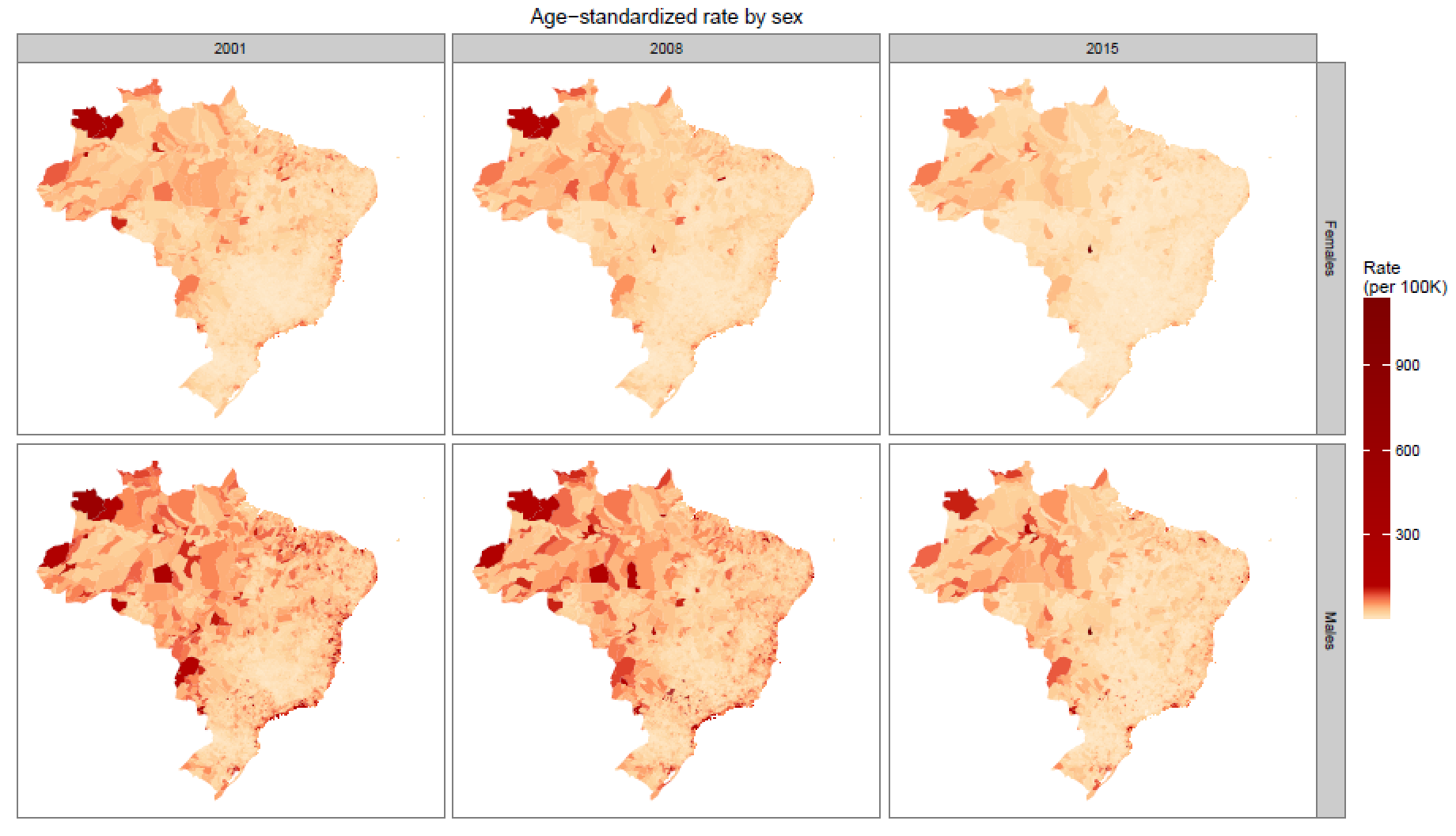


Fig S7: Age-standardised national TB case fatality ratios by year and sex, 2001-2014


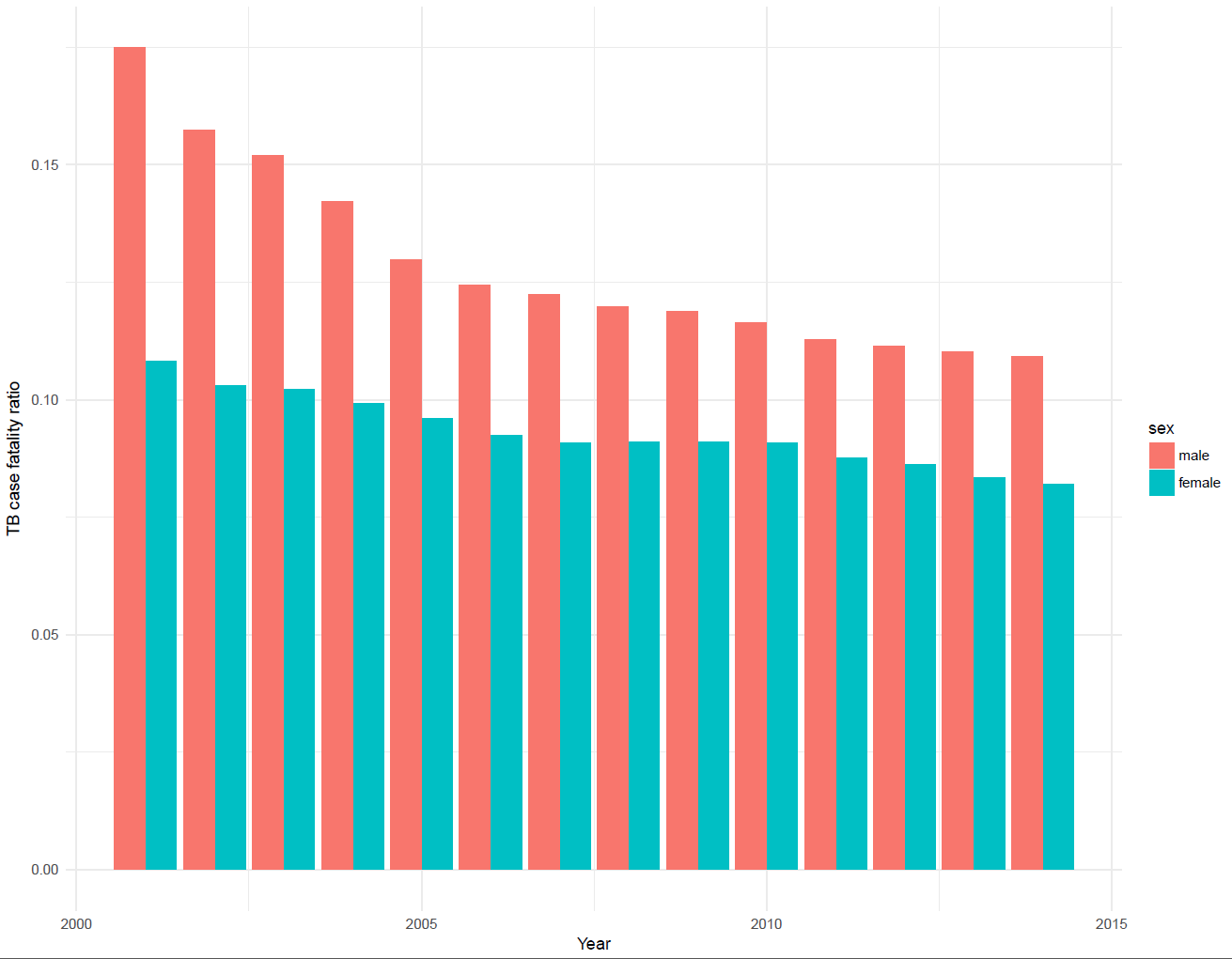


# 5.0 Supplementary References

[1] A. J. Tatem, “WorldPop, open data for spatial demography,” *Sci. Data*, vol. 4, p. 170004, 31 2017.

[2] Food and Agriculture Organization of the United Nations, *Global Administrative Unit Layers (GAUL) (GeoLayer).* 2015.

[3] GBD 2016 Causes of Death Collaborators, “Global, regional, and national age-sex specific mortality for 264 causes of death, 1980-2016: a systematic analysis for the Global Burden of Disease Study 2016,” *Lancet Lond. Engl.*, vol. 390, no. 10100, pp. 1151–1210, Sep. 2017.

[4] K. Kristensen, A. Nielsen, C. W. Berg, H. Skaug, and B. M. Bell, “**TMB** : Automatic Differentiation and Laplace Approximation,” *J. Stat. Softw.*, vol. 70, no. 5, 2016.

[5] R Core Team, “R: a language and environment for statistical computing,” Foundation for Statistical Computing, Vienna, Austria, 2015.

[6] T. Srebotnjak, A. H. Mokdad, and C. J. Murray, “A novel framework for validating and applying standardized small area measurement strategies,” *Popul. Health Metr.*, vol. 8, p. 26, Sep. 2010.

[7] L. Dwyer-Lindgren *et al.*, “US County-Level Trends in Mortality Rates for Major Causes of Death, 1980-2014,” *JAMA*, vol. 316, no. 22, pp. 2385–2401, 13 2016.
